# Dental Wear: A Scanning Electron Microscope Study

**DOI:** 10.1155/2014/340425

**Published:** 2014-12-07

**Authors:** Luca Levrini, Giulia Di Benedetto, Mario Raspanti

**Affiliations:** Department of Surgical and Morphological Sciences, Oro Cranio Facial Disease and Medicine Research Centre, Insubria University, 21100 Varese, Italy

## Abstract

Dental wear can be differentiated into different types on the basis of morphological and etiological factors. The present research was carried out on twelve extracted human teeth with dental wear (three teeth showing each type of wear: erosion, attrition, abrasion, and abfraction) studied by scanning electron microscopy (SEM). The study aimed, through analysis of the macro- and micromorphological features of the lesions (considering the enamel, dentin, enamel prisms, dentinal tubules, and pulp), to clarify the different clinical and diagnostic presentations of dental wear and their possible significance. Our results, which confirm current knowledge, provide a complete overview of the distinctive morphology of each lesion type. It is important to identify the type of dental wear lesion in order to recognize the contributing etiological factors and, consequently, identify other more complex, nondental disorders (such as gastroesophageal reflux, eating disorders). It is clear that each type of lesion has a specific morphology and mechanism, and further clinical studies are needed to clarify the etiological processes, particularly those underlying the onset of abfraction.

## 1. Introduction

Dental wear is a term referring to a group of commonly observed dental tissue disorders, namely, erosion, attrition, abrasion, and abfraction [1]. Although these conditions are all characterized by loss of mineralized tissue, unrelated to bacterial action, they show morphological and etiological differences [2]. Since these different types of lesion often appear concurrently, they can be difficult to identify; for this reason many researchers think that the specific terms erosion, attrition, abrasion, and abfraction should be abandoned in favour of a more generic term such as noncarious cervical lesions (NCCLs) [3]. All dental wear is either mechanical or chemical. Abrasion, attrition, and abfraction fall into the category of mechanical wear, while erosion is chemical wear. Erosion is the gradual but irreversible dissolution of dental tissue caused by acidic agents [4]. Its causes are closely connected with lifestyles, habits, and disorders that are common nowadays and whose identification is crucial in order to establish a correct treatment plan, which may involve not only the dentist but other specialists as well. The main causes of erosion can be divided into extrinsic and intrinsic factors [5]. The former include intake of acid vitamin C supplements [6] and the frequent consumption of acid foods and drinks (fruit juices, citrus fruits, soft drinks, etc.) [7]. The intrinsic factors include gastroesophageal reflux [8], eating disorders such as bulimia and anorexia, and conditions such as pregnancy and obesity that, causing increased gastric pressure, lead to acid reflux. Erosion lesions of the teeth have distinctive rounded edges; furthermore, thanks to the protection afforded by crevicular fluids, part of the enamel on the cervical margins is typically preserved [9, 10]. Attrition lesions present as large, shiny areas with clear margins; attrition defects match the morphology of the opposite teeth. Attrition wear is caused by excessive functional or parafunctional forces [11] and bruxism [12, 13]. Usually, with aging, there is some physiological wear of the tooth substance caused by normal masticatory activities, but beyond a limited threshold this becomes pathological [14]. Abrasion, on the other hand, is due to the interaction between teeth and exogenous objects and substances, such as toothpicks, dental floss, highly abrasive toothpastes (in particular those with a whitening action), pipes, and toothbrushes (especially when these have hard bristles and too much pressure is applied when brushing) [11]. It can also be caused by the abrasive action of foods, for example, vegetables that have not been thoroughly washed can bring traces of soil into contact with the teeth, a problem that can be associated particularly with vegan/vegetarian diets [15]. Abrasion and abfraction are often associated with loss of the soft tissue adjacent to the teeth and they can be accompanied by wedge-shaped defects that are deeper than wider [16]. Abfraction, a term introduced by Grippo in 1991, occurs when the tooth is subjected to extreme stress and fatigue in an area far from the force application point during occlusal and parafunctional loading. Abfraction manifests itself at the cervical margin of the teeth (where they are already weaker because of a thinner prismatic layer) with microfractures and microstructural loss [17]. The study of abfraction is still ongoing; current data are, in some cases, conflicting and incomplete and the biomechanical mechanisms of these cervical lesions remain to be clarified.

The objectives of the dentist faced with any kind of NCCL are dental tissue preservation, aesthetic restoration, and prevention (based on appropriate patient advice).

In these complex disorders, whose prevention and care can involve several specialists, scanning electron microscopy (SEM) examination is an approach that promises to make a major positive contribution, allowing different lesion types to be visualized and providing information that exceeds the data obtained with other instruments. The aim of the present study was to analyze the morphological characteristics and the microwear features of a collection of extracted human teeth showing NCCLs, clarifying their clinical and diagnostic presentations and possible significance.

## 2. Materials and Methods

An expert clinician, using an appropriate chart, analyzed consecutively 250 human teeth extracted in the course of routine dental treatment from patients attending the Ospedale di Circolo, Fondazione Macchi, in Varese (Italy). An initial inspection of these teeth allowed 20 to be picked out as affected by dental wear; 12 of these were then selected because they displayed wear lesions that, thanks to their macroscopic characteristics, could be diagnosed beyond doubt by visual examination, according to definitions of wear lesions reported in the main scientific literature [4, 11, 17]. The selected teeth (three for each wear category) comprised five incisors, two canines, and five premolars. The teeth were stored in 0.5% sodium hypochlorite and copper for decontamination. Taking care not to damage lesion area, the teeth were reduced in size (both breadthwise and lengthwise) to better fit the SEM stub. This was done using a diamond bur handpiece. To prevent inorganic and organic remains and tartar from obstructing the SEM view, the tooth stumps were washed with hydrogen peroxide [18], air dried, mounted on standard SEM stubs using a conductive adhesive, and gold coated using an Emitech K-250 sputter-coater. All the specimens were then observed on an FEI XL-30 FEG (FEI, Eindhoven, The Netherlands) high-resolution SEM fitted with an Everhart-Thornley detector for secondary electrons and a coaxial detector for backscattered electrons. All micrographs were directly obtained in digital form as 1424-by-968 pixel, 8bpp TIFF files. For each lesion the location and shape of the defect and the microwear features of the enamel, dentin, dentinal tubules, and pulp were analyzed and photographed at a magnification ranging from 25x to 5000x.

## 3. Results

**Figure 1:**
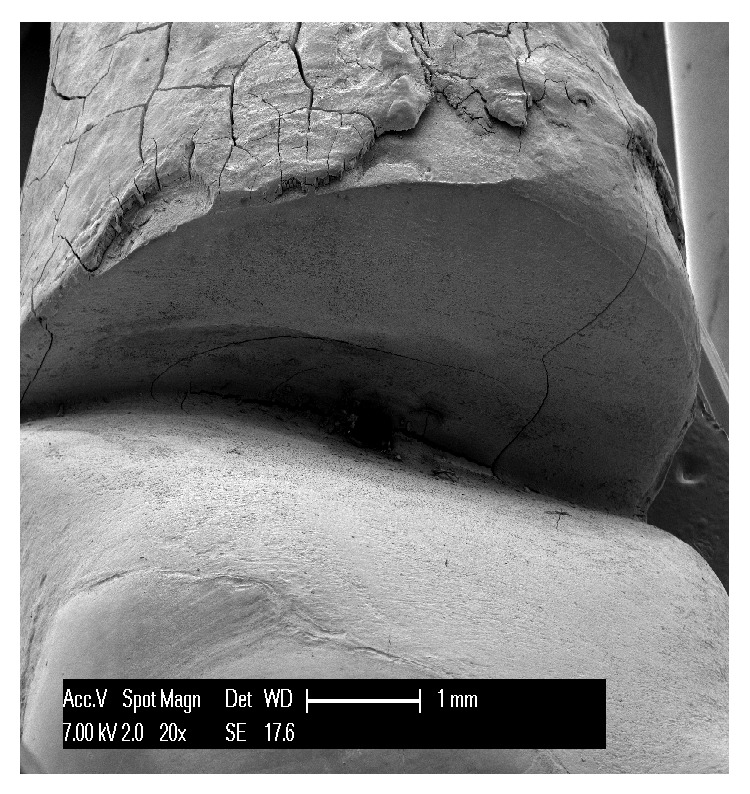
A mandibular premolar showing a typical deep, wedge-shaped abfraction lesion with a roof and a top that both converge towards the pulp forming a sharp internal angle.

**Figure 2:**
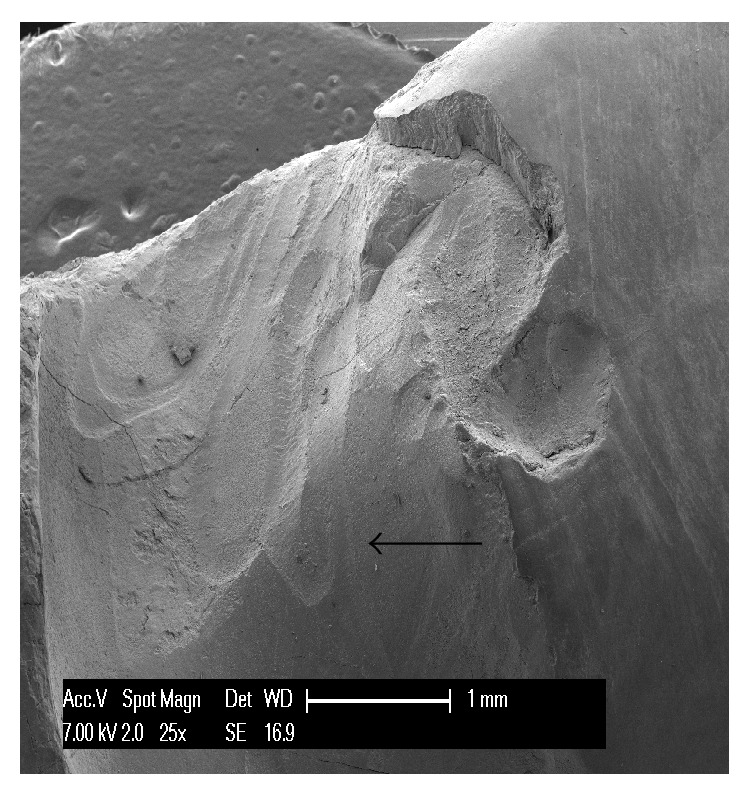
An example of abfraction lesion in an upper canine. The lesion is characterized by multiple cavities and partially overlapping furrows. It is possible to observe parallel striations alternating with protruding crests; a process of mechanical abrasion is superimposed on the abfraction lesion. Although forces propagating from the force application point can be expected to run along the path of least resistance, that is, along the dentinal tubules, this is not what is observed in the generation of abfraction lesions.

**Figure 3:**
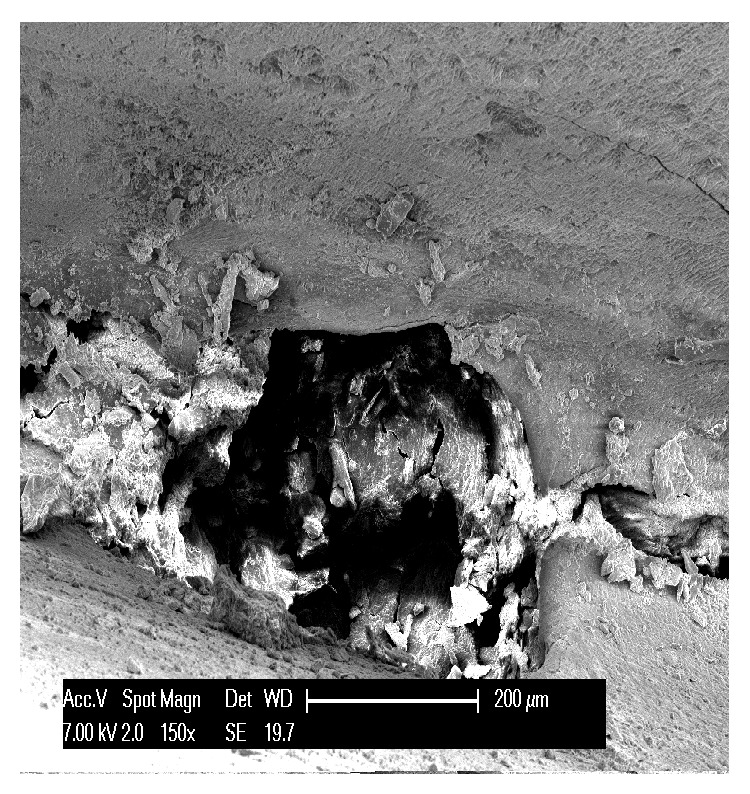
Traces of pulp tissue on the tooth surface.

The first teeth analyzed were the three affected by abfraction. In all three specimens the lesions involved the vestibular cervical margin. At low magnification, they generally appeared wedge-shaped and irregular, with clearly defined edges; their depth was greater than their width (Figure 1). One of the specimens had multiple cavities, which was an unusual feature (Figure 2). In all three specimens, both the top, or ceiling, of the abfraction (at coronal level) and its floor (base) were found to converge towards the pulp, often forming a sharp internal angle (Figure 1). In the advanced stages, as the lesion becomes deeper, the pulp becomes exposed as can be noted by the presence of organic material on the tooth surface (Figure 3). Higher SEM magnifications showed that, in abfraction, all the main components of the tooth were damaged even when the enamel near the lesion was normal and the cementoenamel junction was intact. A smear layer covered the surface of the dentin and the characteristic tubules were not visible. The surface of the cavity could appear smooth or scratched, because abfraction can be accompanied by mechanical abrasion or chemical erosion. Some areas of the lesions, possibly the most recent ones, showed numerous dentinal tubules while others (possibly older) had fewer tubules and a smear layer covering the dentin.

**Figure 4:**
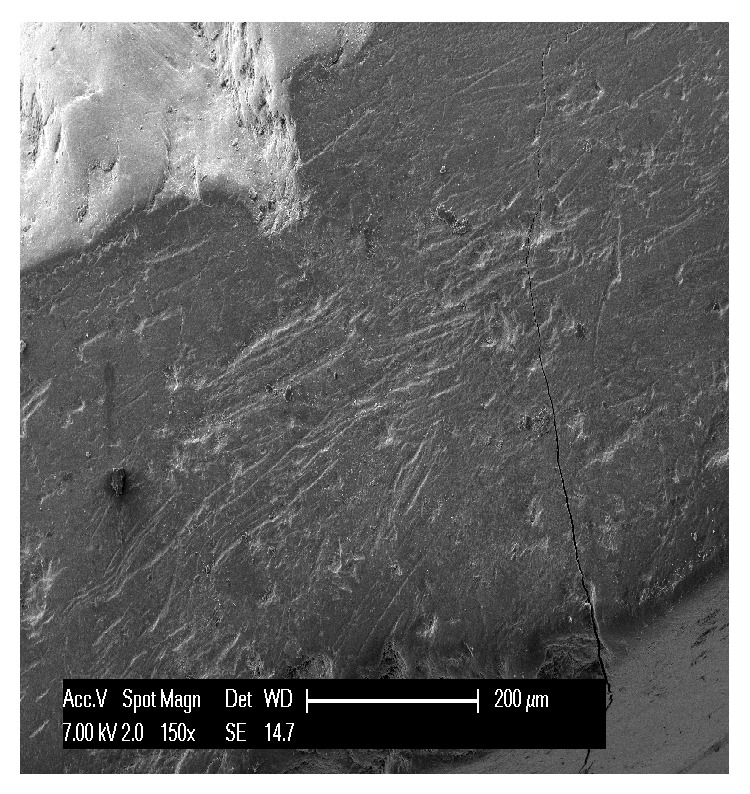
Scratches, stripes, and striations cover the surface of enamel affected by attrition.

**Figure 5:**
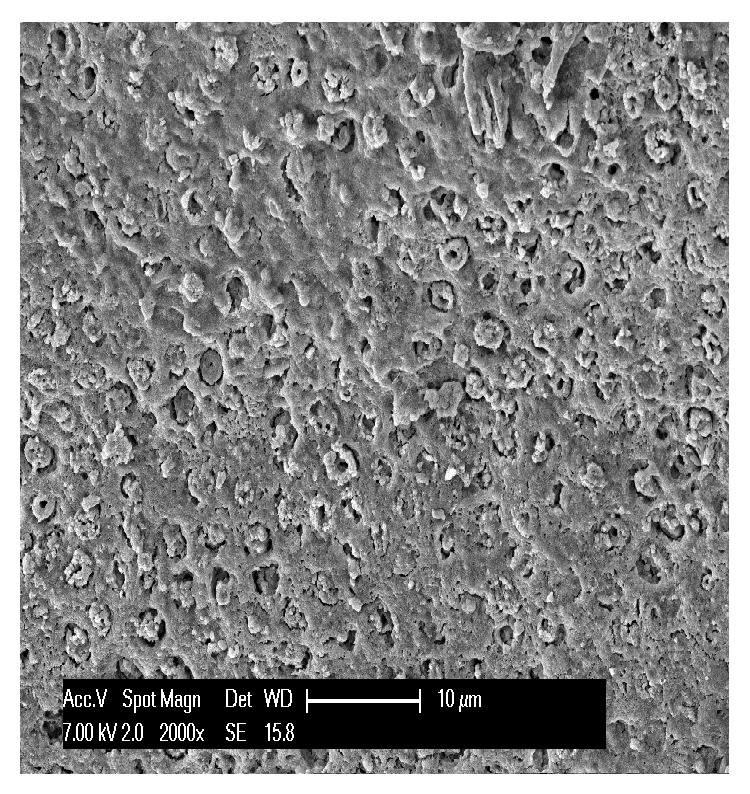
In advanced lesions, there is clear dentin exposure with remains of the smear layer on the surface.

**Figure 6:**
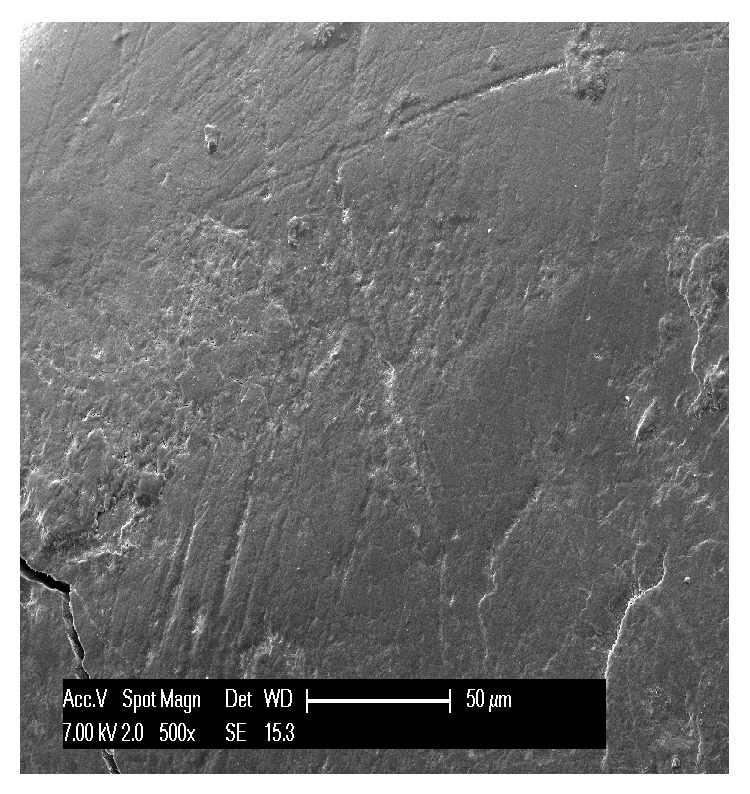
Microscopically visible furrows of variable sizes and fine scratch marks.

Second, we analyzed the three teeth affected by attrition. Defects were found on the occlusal side of the specimens, but also on the vestibular and palatal sides in advanced lesions. At low magnification, this kind of wear appeared more regular and less varied. Attrition seemed to result in a sort of leveling of the cusp and of the incisor margins, with shiny wear facets initially limited to the enamel and subsequently becoming deeper, to reach the pulp. Microscopically, the enamel surface appeared smooth and polished with stripes and striations compatible with protrusion and lateral occlusal movements (Figure 4); it also showed many furrows of variable sizes and fine scratch marks (Figure 6). The enamel prisms were irregular and showed a straight rather than a fan shape arrangement. The dentinal tubules appeared normal, as the dentin is involved only minimally in early-stage lesions. The advanced lesions showed clear dentin exposure with remains of the smear layer on the surface (Figure 5) and in some cases pulp exposure. The exposed pulp had an irregular appearance with areas of resorption as well as areas characterized by substance deposits compatible with the presence of mineralized tissue.

**Figure 7:**
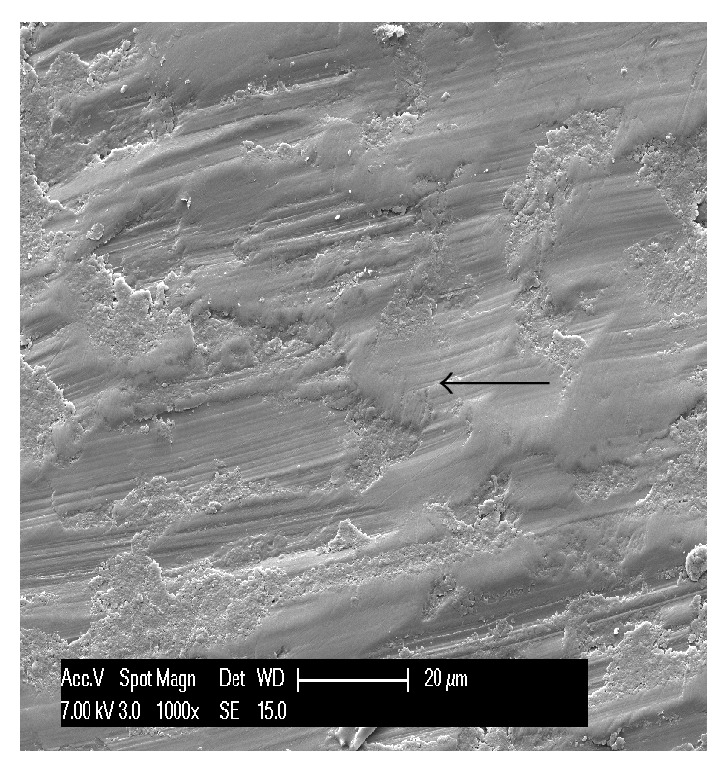
The abraded surface, appearing compressed, shows none of the characteristic features of enamel and dentin.

**Figure 8:**
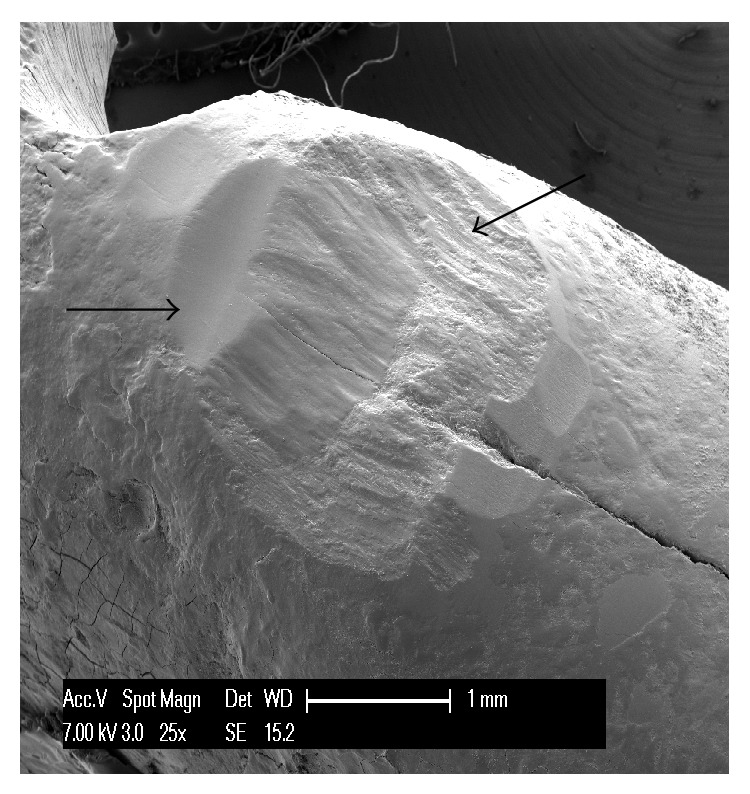
A representative tooth affected by abrasion (on the vestibular side of the lower incisor). The smooth enamel shows striations of different depths, longitudinal and transverse grooves, scratches in lesions caused by delicate pressure, and pitting in ones caused by more intense pressure.

**Figure 9:**
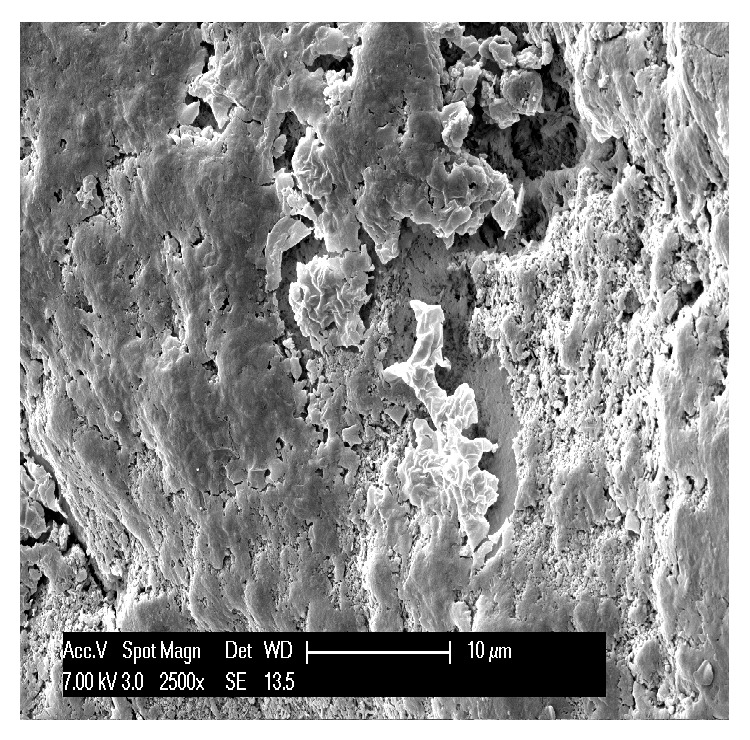
In advanced lesions the dentin is covered with crystallized debris.

The third teeth examined were the ones showing abrasion. This was found on the occlusal side and on the external side of the teeth, depending on the nature and origin of the exogenous traumatizing agent. At low magnification, the lesions were seen as areas clearly distinct from the adjacent healthy tissue and were flat or concave depending on the exogenous abrasive agent; the width of the defect was always greater than its depth. At higher magnifications, the wear surface appeared compressed and it was not possible to identify any of the characteristic features of enamel and dentin (Figure 7). The enamel appeared smooth, with striations of different depths, longitudinal and transverse grooves, scratches in lesions caused by delicate pressure (Figure 8), and pitting in ones caused by more intense pressure. The worn enamel was usually clearly distinct from the healthy enamel although in some samples the enamel near the abrasion showed various abnormalities: the characteristic enamel prisms were not visible and the enamel was covered with inorganic material. In some areas the enamel was intact but the dentin beneath showed multiple fractures. The dentin had few dentinal tubules, which were patent, and was covered with crystallized debris (Figure 9). In the third specimen, different areas of the same vestibular side of the tooth showed evidence of different abrasive agents; variations in their morphology made these areas identifiable under the SEM: some areas were flat, some presented rough scratches, and some showed fine striations; moreover this tooth had no wear facets, only nonanatomically specific wear areas (Figure 8).

**Figure 10:**
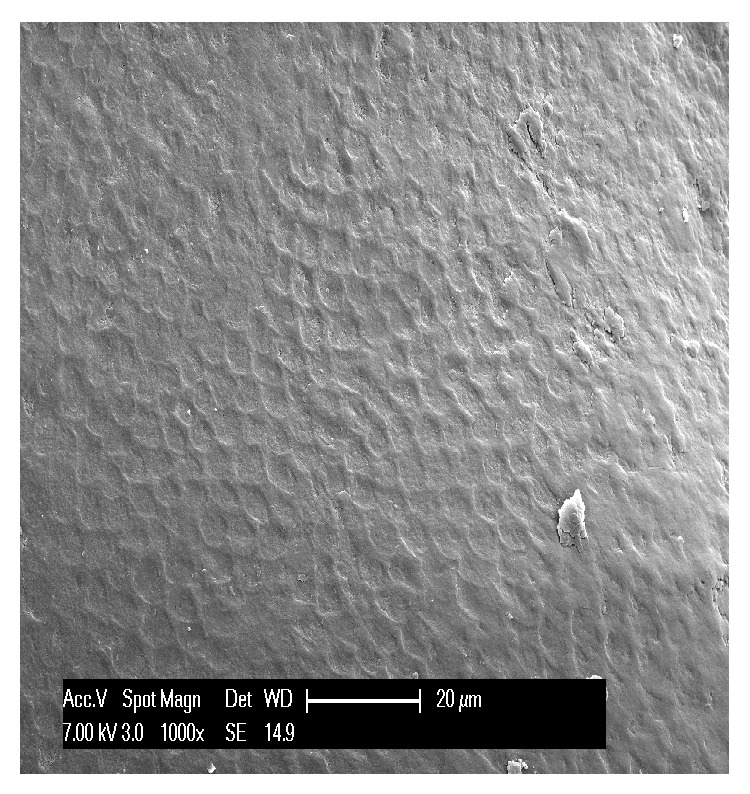
Close-up of chemically eroded enamel displaying the typical honeycomb structure that appears when only the prismatic enamel is eroded, leaving the interprismatic enamel protruding.

**Figure 11:**
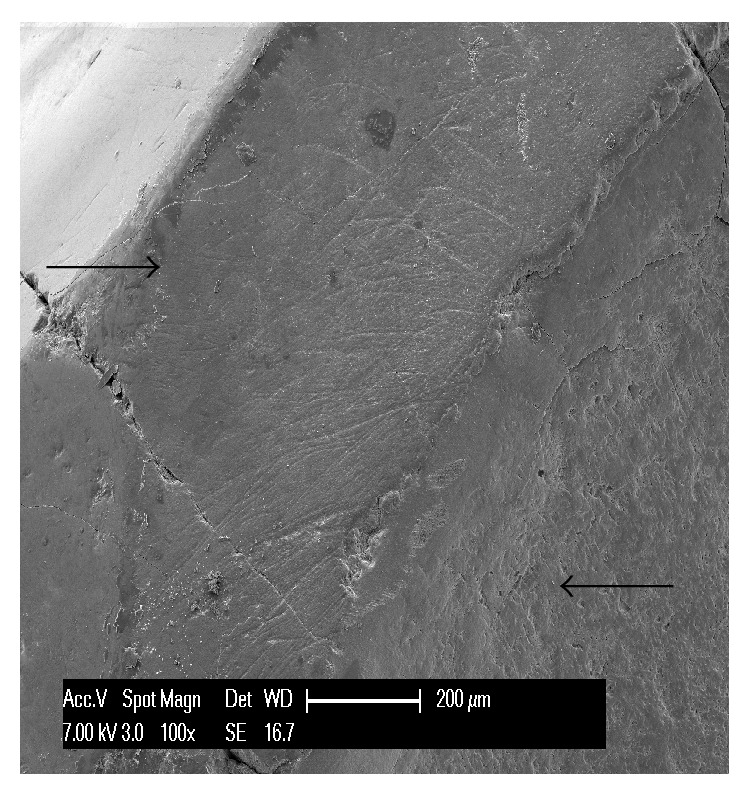
Erosion of a lower premolar that also displays enamel abrasion. The surface shows numerous striations and scratch marks.

**Figure 12:**
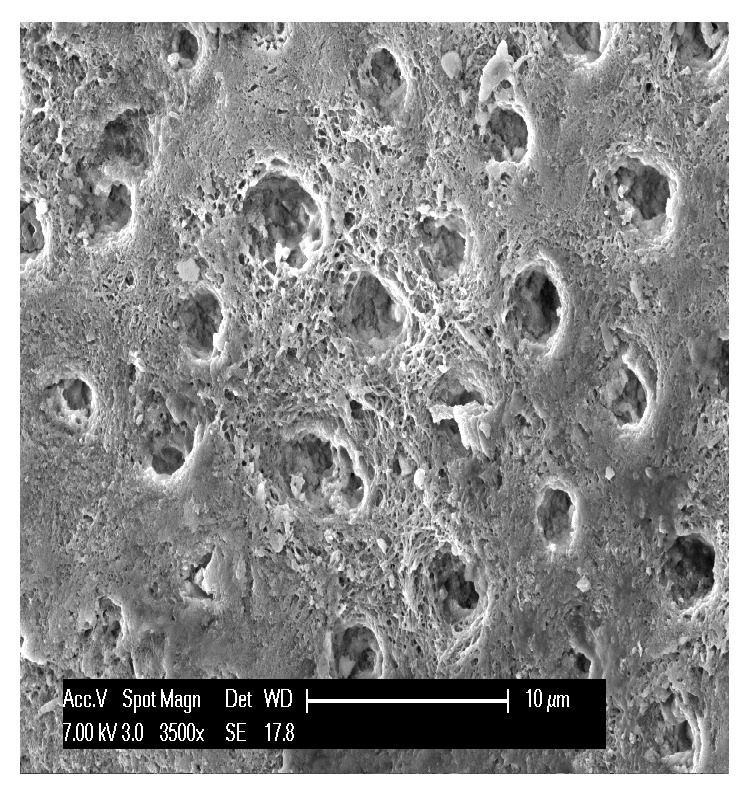
In erosion lesions, the tubules have rounded openings; the collagen matrix between them is eroded and demineralized. In advanced lesions the dentinal tubules are closed and this may eliminate the sensitivity that characterizes early-stage erosion lesions, in which they are still open.

The last three teeth analyzed were the ones showing erosions. At low magnification the eroded enamel appeared smooth and dull, especially in the most vulnerable areas; the areas most exposed to erosion include the internal sides of upper incisors and the vestibular sides of lower posterior teeth. Some healthy enamel frequently remained on the vestibular/palatal cervical margin. Microscopically, the enamel showed a distinctive honeycomb structure, which is due to the fact that chemical erosion affects the prismatic enamel but leaves the interprismatic enamel protruding (Figure 10). Since eroded enamel can be unable to withstand occlusal loading, the erosion could also be accompanied by attrition and abrasion, depending on the type of stress exerted on the teeth (Figure 11). In the advanced lesions there was also pulp involvement, that is, with pulp exposure and the presence of organic material in the site. The dentin at the level of the defect showed the pitting typically associated with acidic attack. In the early-stage lesions the dentinal tubules were patent, whereas they were closed in the advanced lesions. The tubules had a rounded opening and the collagen matrix between the tubules was eroded and demineralized (Figure 12).

## 4. Discussion

The findings herein reported show that SEM studies can be useful for clarifying the main properties of the different types of dental microwear and therefore help clinicians to promptly recognize them.

All the examined lesions were characterized by* dental tissue loss* unrelated to the action of bacteria. Enamel and dentin loss can involve less or more than 50% of the surface area; instead, as regards the pulp, there are three possible types of involvement: the pulp may be visible through the dentin, the pulp chamber may be open in a limited and localized area, or the pulp chamber may be totally open.

Each lesion has a* specific location* on the external (palatal, vestibular, mesial, and distal) and/or occlusal side. On the basis of the analyzed specimens, we can affirm that the external location is typical of abfraction lesions, especially at cervical level; attrition lesions are usually located on the occlusal sides of posterior teeth and on the external sides of anterior teeth; abrasion lesions can appear anywhere, depending on the origin of the traumatizing agent, while erosion lesions are generally found on the occlusal and vestibular surfaces of posterior lower teeth, because, as explained by Daley and colleagues, these areas are lubricated by mucus from the minor salivary glands that does not have the same buffering power as saliva (since saliva protects against these lesions they are uncommon in areas near to the opening of the salivary glands, i.e., on the vestibular side of posterior upper teeth and on the lingual side of the lower incisors) [19]. From the analyzed specimens we can also state that abrasion, erosion, and attrition are not found exclusively at cervical level (indeed, the term NCCLs covers lesions that may also have a different location); however when they are located at cervical level they allow us to make a differential diagnosis.

The* shape of the defect* is a feature that varies between wear lesions, but also between different stages (early or advanced) of the same lesion. They can be flat or concave, irregular (rough, with a number of cavities), and wedge-shaped; they can show pitting, striations, scratches, and enamel cracks and have clearly defined or poorly defined edges. From the analyzed specimens, we can say that abfractions are usually wedge-shaped, irregular lesions, with clearly defined edges, and that they are deeper than they are wide. Their internal angle can be sharp or more rounded. The size of the wedge defect is proportional to the intensity and frequency of the forces applied [20]. In accordance with the defects examined in our study, many researchers describe three types of abfraction: C-shaped lesions with rounded floors, V-shaped lesions, and mixed-shaped lesions with flat cervical and semicircular occlusal walls [21]. It is difficult to understand how a load far from the defect site can be considered to be the cause of these large, heterogeneous cavities. In our three specimens we observed wedge-shaped lesions with flat, regular walls; one of the specimens showed partially overlapping furrows, as shown in Figure 2, with some of these having an irregular surface. These features, in our view and contrary to common opinion, seem to indicate that they are caused by a direct impact on the area involved. In fact, forces propagating from the application point can be expected to run along the path of least resistance, that is, along the dentinal tubules; however, this is not what is observed in the generation of abfraction lesions.

In the first stages of attrition, when the wear is limited to the enamel, the facets are smooth and flat, with clearly defined edges; in the advanced stage, on the other hand, they become concave because the dentin is exposed and wears more rapidly than the enamel does. Like us, Kaidonis (2008) describes attrition characterized by distinctive parallel striations. The lesion is considered to be active when the facets are well defined and shiny [11].

An abrasion lesion is flat or concave, depending on the exogenous abrasive agent. The width of the lesion is greater than its depth. Various studies describe scratch marks, striations, and pitting, which reflect the effects, on the dental surfaces, of foods or other forces [11]. The depth and length of the scratches reflect the kind of diet consumed. Moreover, contrary to what is seen in erosion, in abrasion the depth/breadth ratio tends to remain constant as the wear progresses [11]. In accordance with the findings of other authors, the erosion lesions present on our specimens took the form of perpetually increasing cavities; in these lesions, the level of sensitivity is determined by the state of the dentinal tubules (open in active lesions) [11]. The SEM results obtained in a study by Daley et al. indicate that dentinal tubules that are open as a result of erosion are repaired by the salivary film or by mineral deposits from saliva. Cervical erosions that are sensitive have open dentinal tubules. In asymptomatic teeth the tubules may instead be closed as a result of the formation of sclerotic dentin, which is similar in its matter and organization to peritubular dentin (cuboidal or rhomboid shaped carbonate crystals associated with a collagen matrix) [19].

Our results confirm those of Nguyen et al. who reported that scratch marks on enamel and dentin are typical of abrasion, while corrosive (erosion) lesions are characterized by smooth surfaces, with the enamel showing a honeycomb structure and the dentin showing a waved or rippled surface. Abrasion and corrosion form scratch marks that are not as noticeable as those caused by abrasion alone [2].

There are also several specific features that facilitate the diagnostic identification of lesions.

- In erosion some healthy enamel is generally preserved on the palatal/vestibular cervical margin; when erosion occurs on the occlusal surfaces, it shows characteristic pitting.
- In attrition, the pattern of tooth substance loss matches the morphology of the teeth in the opposing arch. Attrition lesions show a well-defined wear facet, with parallel striations and stripes which are compatible with protrusion and lateral occlusal movements at the level of the facet border.
- Abrasion lesions do not show wear facets, but nonanatomically specific wear areas (as illustrated by comparison of Figures 4 and 8) and they are usually associated with gingival and periodontal recession.
- Abfraction usually takes the form of a wedge-shaped lesion located at cervical level.

In line with the findings reported herein and the lesion surface shown in Figure 7, Karan et al. and colleagues, in a more detailed study, described alterations of the matrix and mineral component that determine the structure and treatment of NCCLs. It could be useful to examine a series of aspects: (i) how the damaged dentin, compared with normal dentin, reacts to acid etching and bonding; (ii) how it might interfere with action of the acid conditioners (penetration and enamel demineralization); (iii) the predictability of the hybridization process; (iv) the degree to which the bond power might become unstable and reduced; and (v) the degree of collagen cross-linking compared with normal dentin [1].

The* number of teeth* affected by wear can vary. It is usually rare to find more than four abfraction lesions in an oral cavity, as well as erosion of just one tooth. As we observed, the number of lesions and their size both increase over time and with patient age [22].

For a correct diagnosis, the clinician, having identified a lesion on the basis of the above-described properties, must compare this data with etiological factors drawn from the patient's medical history. These factors are divided into two categories: extrinsic and intrinsic. The following causative factors are extrinsic in nature: acid foods (related to erosion), reduced salivary flow (erosion), drugs that change the buffering power of saliva (erosion), traumatizing agents such as pipes, dental floss, and toothbrushes (associated with abrasion), poorly washed vegetables (abrasion), and immune system disease (erosion). The following agents belong to the intrinsic category: parafunctions (attrition, abfraction), gastroesophageal reflux (erosion), eating disorders (erosion), bruxism (attrition), and abnormal occlusal or biomechanical loads/forces (abfraction) [4, 13, 17].

The information set out in this paper should help dentists to recognize the different wear lesion presentations and thus to improve the prevention of dental wear (through the imparting of appropriate advice to patients with etiopathological risk factors) and to plan treatments correctly. Indeed, whereas diagnostic agreement has been reported to be 99% for carious cervical lesions, it is just 38–49% for NCCLs [23]. The diagnosis is frequently complicated by the concomitance of different types of defect; their etiology, too, can be multifactorial [24].

## 5. Conclusions

This report, based on SEM study and analysis of a series of extracted human teeth, provides a complete overview of the distinctive morphological characteristics and the microwear features of dental wear lesions, clarifying their clinical and diagnostic presentations and possible significance. Our findings, even though they are based on a limited number of specimens, consistently provide evidence that is in line with current knowledge. They allow us to affirm that dental wear, partly because different types of lesion can be present concomitantly, can take many forms; it is important to identify each type of lesion in order to recognize the contributing etiological factors and, consequently, identify other more complex, nondental disorders (e.g., gastroesophageal reflux, eating disorders). It is clear that each kind of lesion has a specific morphology and mechanism, and further clinical studies are needed to clarify the etiological processes, particularly those underlying the onset of abfraction, which is still a controversial topic. Further research is needed to provide the basis for a deeper understanding of this phenomenon and long-term clinical studies based on a large number of samples are needed to improve the treatment of dental wear defects generally, particularly as regards the response of these altered substrates to acid etching and bonding.

## References

[1] Karan K., Yao X., Xu C., Wang Y. (2009). Chemical profile of the dentin substrate in non-carious cervical lesions. *Dental Materials*.

[2] Nguyen C., Ranjitkar S., Kaidonis J. A., Townsend G. C. (2008). A qualitative assessment of non-carious cervical lesions in extracted human teeth. *Australian Dental Journal*.

[3] Telles D., Pegoraro L. F., Pereira J. C. (2000). Prevalence of noncarious cervical lesions and their relation to occlusal aspects: a clinical study. *Journal of Esthetic Dentistry*.

[4] Johansson A.-K., Omar R., Carlsson G. E., Johansson A. (2012). Dental erosion and its growing importance in clinical practice: from past to present. *International Journal of Dentistry*.

[5] Ganss C., Klimek J., Giese K. (2001). Dental erosion in children and adolescents—a cross-sectional and longitudinal investigation using study models. *Community Dentistry and Oral Epidemiology*.

[6] Meurman J. H., Murtomaa H. (1986). Erosion due to vitamin C tablets. *Tandlakartidningen*.

[7] Lussi A., Jaeggi T. (2006). Dental erosion in children. *Monographs in Oral Science*.

[8] Holbrook W. P., Furuholm J., Gudmundsson K., Theodórs A., Meurman J. H. (2009). Gastric reflux is a significant causative factor of tooth erosion. *Journal of Dental Research*.

[9] Lussi A., Jaeggi T., Schaffner M. (2004). Prevention and minimally invasive treatment of erosions. *Oral Health & Preventive Dentistry*.

[10] Lussi A., Jaeggi T., Zero D. (2004). The role of diet in the aetiology of dental erosion. *Caries Research*.

[11] Kaidonis J. A. (2008). Tooth wear: the view of the anthropologist. *Clinical Oral Investigations*.

[12] Krogh-Poulson W., Carlsen O. R. (1979). *Bidfunktion/Bettfysiologi*.

[13] Dahl B. L., Krogstad O., Karlsen K. (1975). An alternative treatment in cases with advanced localized attrition. *Journal of Oral Rehabilitation*.

[14] Robb N. D. (1992). *Epidemiological study of tooth wear [Ph.D. thesis]*.

[15] Smith B. G. (1989). Toothwear: aetiology and diagnosis. *Dental Update*.

[16] Smith B. G., Knight J. K. (1984). A comparison of patterns of tooth wear with aetiological factors. *British Dental Journal*.

[17] Grippo J. O. (1991). Abfractions: a new classification of hard tissue lesions of teeth. *Journal of Esthetic Dentistry*.

[18] Lolayekar N. V., Bhat S. V., Bath S. S. (2007). Disinfection methods of extracted human teeth. *Journal of Oral Health and Community Dentistry*.

[19] Daley T. J., Harbrow D. J., Kahler B., Young W. G. (2009). The cervical wedge-shaped lesion in teeth: a light and electron microscopic study. *Australian Dental Journal*.

[20] Lee W. C., Eakle W. S. (1984). Possible role of tensile stress in the etiology of cervical erosive lesions of teeth. *The Journal of Prosthetic Dentistry*.

[21] Hur B., Kim H.-C., Park J.-K., Versluis A. (2011). Characteristics of non-carious cervical lesions—an ex vivo study using micro computed tomography. *Journal of Oral Rehabilitation*.

[22] Wood I., Jawad Z., Paisley C., Brunton P. (2008). Non-carious cervical tooth surface loss: a literature review. *Journal of Dentistry*.

[23] Bader J. D., Levitch L. C., Shugars D. A., Heymann H. O., McClure F. (1993). How dentists classified and treated non-carious cervical lesions. *The Journal of the American Dental Association*.

[24] Litonjua L. A., Andreana S., Bush P. J., Tobias T. S., Cohen R. E. (2003). Noncarious cervical lesions and abfractions: a re-evaluation. *Journal of the American Dental Association*.

